# ACSS2 is required for colorectal cancer progression and a druggable target for colorectal cancer treatment

**DOI:** 10.1016/j.isci.2026.116860

**Published:** 2026-07-21

**Authors:** Lei Wang, Urszula Dougherty, Wenliang He, Lu Gao, Caleb Muefong, Rajesh Sarkar, Jie Du, Ardaman Shergill, Hening Lin, Marc Bissonnette, Yan Chun Li

**Affiliations:** 1 Department of Medicine, Division of Biological Sciences, The University of Chicago, Chicago, IL, USA; 2 Department of Chemistry, Division of Biological Sciences, The University of Chicago, Chicago, IL, USA; 3 Howard Hughes Medical Institute, The University of Chicago, Chicago, IL, USA; 4 Committee on Molecular Metabolism and Nutrition, The University of Chicago, Chicago, IL, USA

**Keywords:** ACSS2, ACSS2 inhibitor, colorectal cancer, acetate, acetyl-CoA

## Abstract

Acyl-CoA synthetase short chain family member 2 (ACSS2) catalyzes the conversion of acetate to acetyl-CoA. Here we show that *ACSS2* expression is markedly elevated in all stages of human colorectal cancer (CRC), and *Acss2* silencing or genetic ablation leads to a marked reduction in CRC tumor load in allograft and xenograft models and CRC models induced by epithelial *Apc* deletion or azoxymethane/dextran sodium sulfate treatment. Tumors with ACSS2 depletion exhibit robust DNA damage, excessive apoptosis, and increased recruitment of macrophages and CD8^+^ T cells to the tumor microenvironment. Treatment with a small-molecule ACSS2 inhibitor markedly suppresses tumor growth in allograft/xenograft and *Apc*-mutant CRC models. ACSS2 promotes tumor cell growth by blocking DNA damage and apoptosis under nutritional stress. Collectively, these data indicate that the conversion of acetate to acetyl-CoA by ACSS2 is required for CRC progression and ACSS2 is a potential druggable target for CRC management.

## Highlights

• ACSS2 is highly upregulated in colorectal cancer
• ACSS2 is required for the progression of colorectal cancer
• Suppression of ACSS2 promotes tumor apoptosis and immune cell infiltration
• ACSS2 is a druggable therapeutic target for the treatment of colorectal cancer

## Graphical abstract

**Figure:**
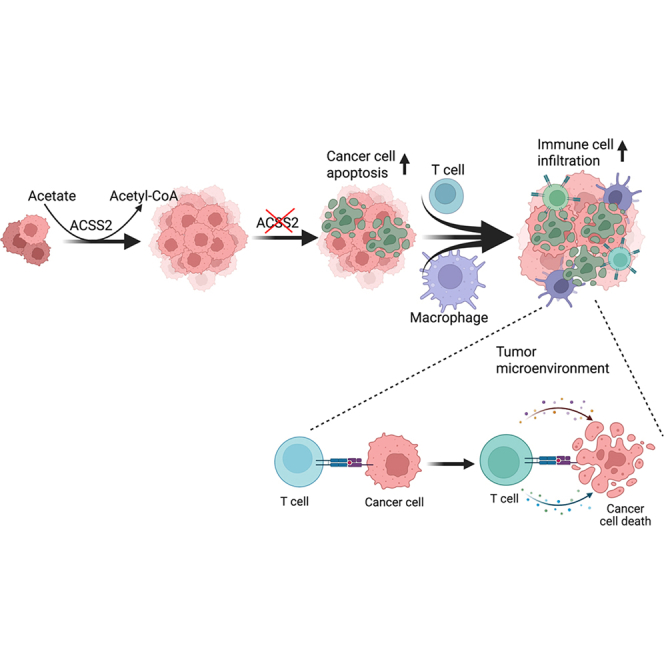

## Teaser

Immunology; Biochemistry; Genetics; Molecular biology; Cell biology

## Introduction

Acetyl-coenzyme A (acetyl-CoA) is a key metabolite in cellular metabolism. It is required for the tricarboxylic acid (TCA) cycle and oxidative phosphorylation in mitochondria, for the *de novo* synthesis of fatty acids and cholesterol, and for protein acetylation. In eukaryotic cells, nucleocytosolic acetyl-CoA is derived mainly from mitochondria-generated citrate. Citrate is a key intermediate in the TCA cycle that can cross mitochondrial membranes to the cytoplasm via the transporter SLC25A1, where it is converted to acetyl-CoA and oxaloacetate by ATP-citrate lyase (ACLY).^1^^,^^2^ Another evolutionally conserved reaction for acetyl-CoA synthesis involves the conversion of acetate to acetyl-CoA, catalyzed by acyl-CoA synthetase short chain family members 1 and 2 (ACSS1, ACSS2). ACSS1 is a mitochondrial enzyme, whereas ACSS2 is located in the cytosolic and nuclear compartments.^3^^,^^4^ Although ACSS2 has long been known to catalyze the conversion of acetate to acetyl-CoA, a recent study reported that this enzyme is also involved in lactyl-CoA synthesis and histone lactylation.^5^ It is known that nutritional stress promotes ACSS2 phosphorylation at S695 via AMP-activated protein kinase (AMPK), leading to ACSS2 nuclear translocation, which increases histone acetylation and upregulates transcription of genes involved in lysosomal biogenesis and autophagy.^6^ Nuclear ACSS2 is also involved in the reuse of acetate released from histone deacetylation to prevent the loss of histone acetylation during hypoxia and serum limitation.^7^ A recent study reports that ACSS2 drives the production of senescence-associated secretory phenotype by limiting purine biosynthesis.^8^ Mice with global *Acss2* deletion are phenotypically normal but exhibit reduced tumor burden in a model of liver cancer.^9^ *Acss2* deletion leads to reduced dietary lipid absorption from the intestine and suppresses the development of hepatic steatosis in a diet-induced obesity model.^10^ Another important physiological function of ACSS2 is to control stress erythropoiesis by facilitating HIF-2α acetylation, thereby activating this hypoxia-responsive transcription factor.^11^

In rapid cell division as occurs during tumor growth, a high demand for nucleocytosolic acetyl-CoA is needed to maintain the synthesis of new lipids and histone acetylation. The latter is critical for chromatin remodeling in gene regulation.^12^ Cancer cells are known to exhibit distinct metabolism.^13^ For example, most malignant cells rely on aerobic glycolysis for rapid energy production and biomass synthesis. In this so-called Warburg effect, pyruvate is converted to lactate without entering the mitochondria for the TCA cycle,^14^ which may inevitably lead to insufficient citrate supply for ACLY-catalyzed cytosolic acetyl-CoA production. This can be exacerbated by glutamine deficiency that is common in solid tumors, because glutamine is used to replenish the TCA cycle in cancer.^15^^,^^16^ As such, highly proliferative tumor cells are thought to depend on extra carbon sources to maintain cellular acetyl-CoA production. One important source could be acetate conversion to acetyl-CoA by ACSS2. In fact, acetate is a bioenergetic substrate for glioblastoma and hepatocellular carcinoma,^9^^,^^17^ and ACSS2 has been shown to promote acetate utilization and maintain cancer cell growth under metabolic stress.^3^ ACSS2 is found to be highly induced in myeloma cells derived from patients with obese and promotes myeloma progression. The underlying mechanism is that ACSS2 facilitates the acetylation of the oncogenic IRF4 protein to stabilize IRF4 for tumorigenesis.^18^ Given the important roles of ACSS2 in tumor metabolism and tumor growth, targeting acetate metabolism by pharmacological agents has been proposed for cancer therapy,^19^ and small molecule inhibitors of ACSS2 have been reported to suppress cancer growth in triple negative breast cancer and myeloma models.^18^^,^^20^

Colorectal cancer (CRC) is the second leading cause of cancer deaths in the U.S.^21^ Because little is known about the role of acetyl-CoA in CRC development, we surveyed the expression of *ACLY*, *ACSS1,* and *ACSS2* in human CRC biopsies in The Cancer Genome Atlas Program (TCGA) database, and found that *ACSS2* is greatly elevated in human CRC. This finding prompted us to investigate the role of ACSS2 in colorectal tumorigenesis. Here we present evidence to demonstrate that ACSS2 is required for the progression of colon cancer, and ACSS2 is a potential druggable therapeutic target for CRC management.

## Results

### ACSS2 is up-regulated in colorectal cancer

**Figure 1:**
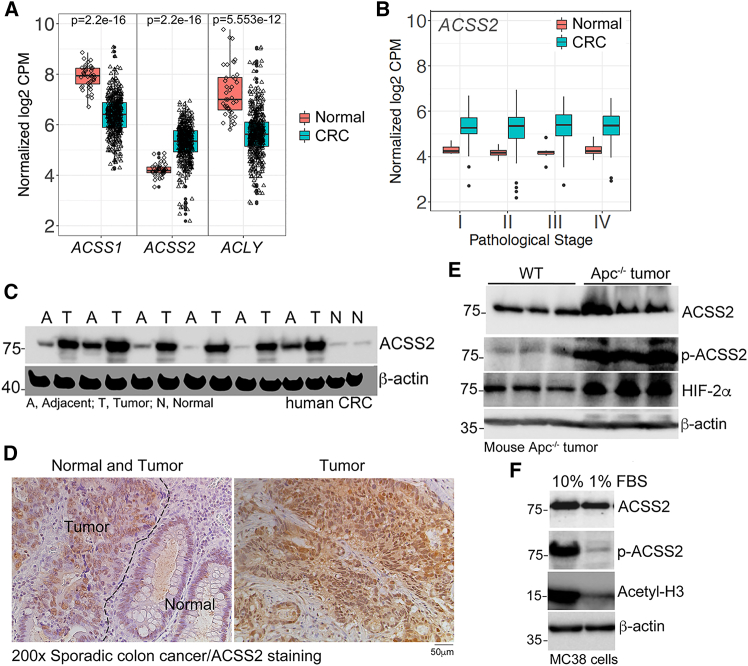
*ACSS2* is up-regulated in colon cancer (A) Expression levels of *ACSS1, ACSS2*, and *ACLY* mRNAs in human colorectal cancer (CRC) and normal colon biopsies. The data were extracted from the TCGA database; (B) *ACSS2* expression levels in stage I to IV human CRC biopsies from the TCGA database; (C) ACSS2 protein levels in human CRC biopsies analyzed by western blotting; T, tumor; A, adjacent normal tissues; N, normal colon tissues without tumor; (D) Immunostaining of ACSS2 expression in human CRC biopsies; *Dash line* separates the tumor and adjacent normal region in the *left* panel; scale bars, 50 μm. (E) Western blot analyses of wildtype (WT) colon samples and *Apc*^−/−^ colon tumors. Each lane represents an independent tumor; (F) Western blot analysis of MC38 cells cultured in 10% or 1% FBS. Statistical analyses were performed by unpaired Welch’s *t* test.

To explore the role of acetyl-CoA in CRC, we mined TCGA database for the expression of the key enzymes involved in acetyl-CoA biosynthesis in human CRC, namely ACSS1, ACSS2, and ACLY. Based on the transcriptomic data from 440 primary colon tumors and 40 normal colon tissues, we found that *ACSS1* and *ACLY* transcript levels are markedly reduced, whereas *ACSS2* levels are markedly elevated, in human CRC (Figure 1A). When stratified by stage of disease, these 440 tumor samples show an up-regulation of the *ACSS2* transcript in all 4 stages of human CRC (Figure 1B). In agreement with its transcript up-regulation, we confirmed by western blotting that ACSS2 protein is dramatically increased in human colon cancers compared to the adjacent normal-appearing colonic mucosa (Figure 1C). Immunostaining data showed clear elevation of ACSS2 in the tumor compared to the adjacent normal colon tissue (Figure 1D, *left panel*), and ACSS2 accumulates in the nucleus of the malignant cells (Figure 1D, *right panel*). We further examined mouse colon cancer samples and confirmed that ACSS2 and HIF-2α (a hypoxia marker) are up-regulated in mouse *Apc*^−/−^ colon tumors (Figure 1E). Interestingly, phosphorylated ACSS2 (*p*-ACSS2), known to be located in the nucleus,^6^ is markedly elevated in these colon tumors (Figure 1E). We also found that MC38 mouse colon cancer cells cultured in 10% fetal bovine serum (FBS) showed increases in ACSS2, *p*-ACSS2, and acetylated H3 histone compared with MC38 cells grown in 1% FBS (Figure 1F), suggesting ACSS2 and ACSS2 phosphorylation are involved in the regulation of colon cancer cell proliferation.

### *Acss2* silencing reduces CRC tumor growth by triggering excessive apoptosis

**Figure 2:**
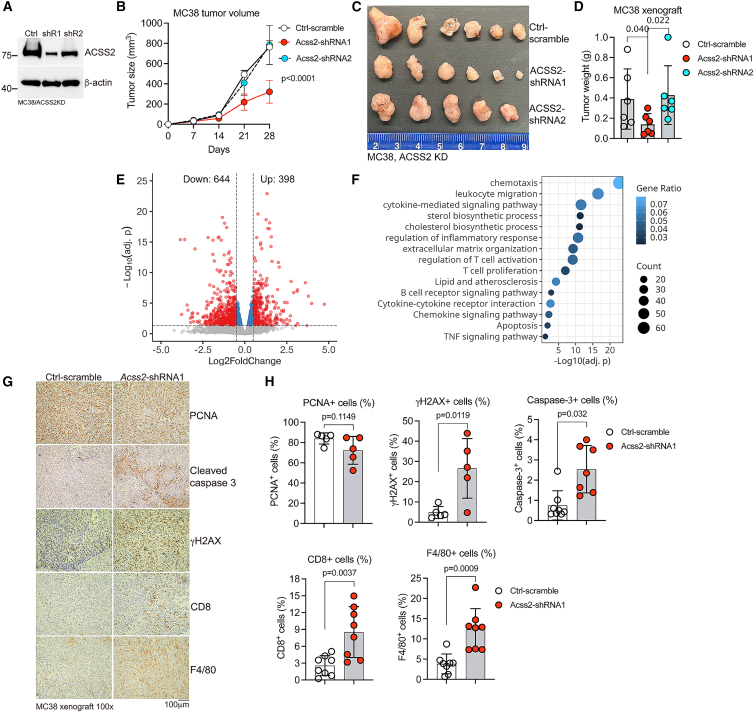
*Acss2* knockdown reduces CRC allograft tumor growth (A) Western blot quantitation of ACSS2 protein levels in MC38 cells stably transduced with scramble RNA control (Ctrl) lentivirus or lentivirus carrying shRNA1 (shR1) or shRNA2 (shR2); (B) Tumor growth curves in mice implanted with MC38 cells transduced with Ctrl, shRNA1 or shRNA2; *n* = 6 each group; (C) Gross images xenograft tumors generated with MC38 cells transduced with Ctrl, shRNA1 or shRNA2; (D) Tumor weight; *n* = 6 each group; (E) Volcano plot of RNA-seq data from Ctrl-MC38 and shRNA1-MC38 xenograft tumors; (F) Top enriched pathways identified in the differentially expressed genes (DEGs) from gene ontology biological process (GO-BP) pathway analysis; (G) Representative images from immunostaining analysis of Ctrl-MC38 and shRNA1-MC38 xenograft tumors; scale bars, 100 μm. (H) Quantitation of the immunostaining data; n = 5–8 each group. Data were presented as mean ± SD. Statistical analyses were performed by unpaired two-way ANOVA or Student’s *t* test.

To address the role of ACSS2 in CRC growth, we assessed the effect of *Acss2* knockdown (KD) on MC38 allograft growth. By lentivirus-mediated shRNA delivery, we obtained different degrees of KD of ACSS2 protein in two stable MC38 clones (shR1 ∼80% KD; shR2 ∼40% KD) using two different *Acss2*-specific shRNAs (Figure 2A). When these clones were used to generate allografts in mice, tumor growth was positively correlated with ACSS2 protein expression levels. The shR1 clone with ∼80% ACSS2 KD showed >50% reduction in tumor growth compared with the scramble RNA control lentivirus (Ctrl)-transduced clone, whereas the shR2 clone with ∼40% ACSS2 KD exhibited no effects on tumor growth (Figures 2B–2D). These results demonstrate a critical role for ACSS2 in CRC tumor progression.

To explore the mechanism whereby ACSS2 KD causes the suppression of tumor growth, we performed bulk RNA-seq analysis to compare the transcriptomic profiles of the tumors derived from the Ctrl clone and the shR1 clone. RNA-seq data revealed 398 up-regulated and 644 down-regulated differentially expressed genes (DEGs) (Figure 2E), and these DEGs are involved in pathways related to chemotaxis, leukocyte migration, inflammation, T and B cell activation, sterol synthesis, and apoptosis (Figure 2F). By immunostaining, we confirmed that the shR1 allograft tumors exhibited marked elevation in γH2AX expression (DNA damage marker) and caspase 3 activation (apoptosis marker) as well as in the accumulation of F4/80^+^ macrophages and CD8^+^ T cells (Figure 2G and 2H). Hypoxia and nutritional stress commonly occur in solid tumors, particularly as the tumor mass increases, and these factors can cause DNA damage leading to p53 activation and apoptosis.^22^^,^^23^ Our observations, therefore, suggest that ACSS2 depletion triggers excessive DNA damage leading to increased apoptosis, which limits tumor growth. On the other hand, increased DNA damage and apoptosis in turn could stimulate local inflammation that recruits macrophages and CD8^+^ T cells to destroy the tumor cells.

### ACSS2 depletion promotes tumor cell apoptosis under nutritional stress

**Figure 3:**
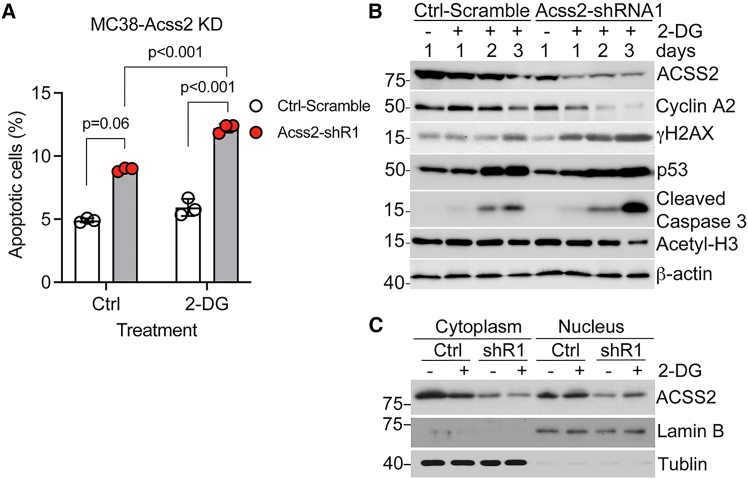
*Acss2* knockdown increases apoptosis in colon tumor cells under nutritional stress (A) Ctrl-MC38 and shRNA1-MC38 cells were cultured in 1% FBS in the presence or absence of 10 mM 2-deoxy-D-glucose (2-DG) for 2 days. The cells were stained with annexin V, and apoptotic cells were quantified by FACS; *n* = 3 each group. (B) Western blot analysis of these cell lysates exposed to 1% FBS and 2-DG for 1, 2, or 3 days; (C) Western blot analysis of cytosolic and nuclear fractions prepared from these cells following exposure to 1% FBS and 2-DG for 2 days. Data were presented as mean ± SD. Statistical analyses were performed by two-tailed Student’s *t* test.

To confirm the notion that ACSS2 protects cancer cells against DNA damage and apoptosis under nutritional stress, we studied Ctrl-scramble RNA- and Acss2-shR1-transduced MC38 cells cultured in a medium containing a low concentration of FBS and 2-deoxy-D-glucose (2-DG), an inhibitor of glycolysis. Compared with Ctrl-MC38 cells, shR1-MC38 cells showed a marked increase in apoptosis within 48 h under the stress of 1% FBS and 2-DG (Figure 3A). Western blot analysis revealed more dramatic increases in γH2AX and p53 expression as well as caspase 3 activation in shR1-MC38 cells in 2-3 days under this condition, accompanied by more dramatic down-regulation of cyclin A2 and H3 histone acetylation (Figure 3B). It is known that under nutritional stress, ACSS2 becomes phosphorylated by AMPK and migrates to the nucleus to promote histone acetylation.^6^ We confirmed that ACSS2 KD led to a decrease in ACSS2 nuclear translocation under this nutritional stress (Figure 3C), which is consistent with the reduction in H3 acetylation seen in shR1-MC38 cells (Figure 3B). These observations suggest that ACSS2 functions to block tumor cell apoptosis in solid tumors via suppressing DNA damage and DNA damage-induced p53 up-regulation, and this probably involves an epigenetic mechanism.

### Genetic ablation of the *Acss2* gene reduces colon tumor burden

**Figure 4:**
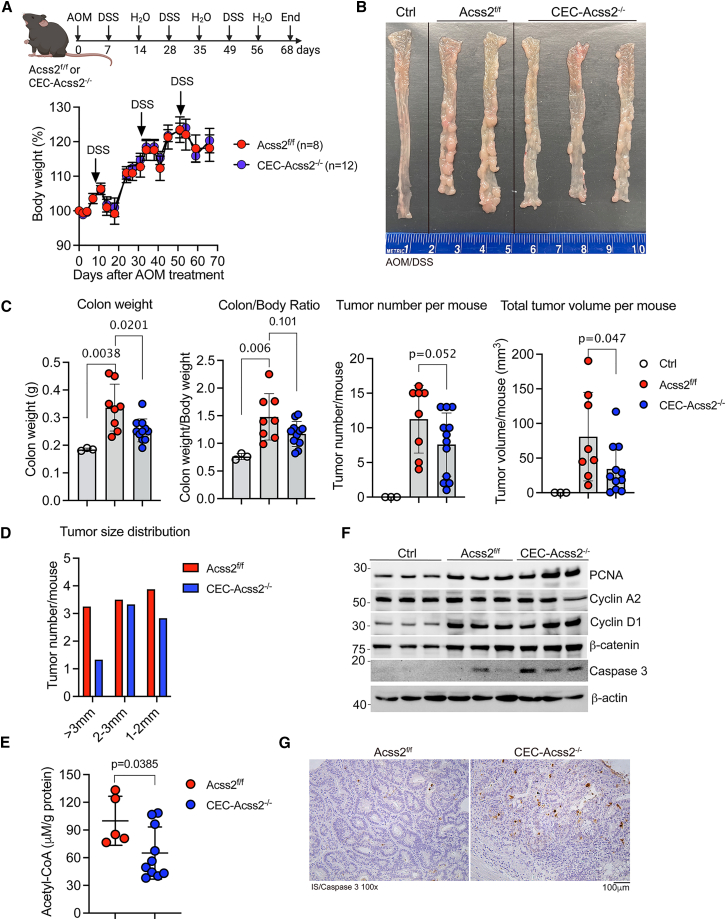
Genetic ablation of the *Acss2* gene reduces colon tumor loads in a colitis-associated colon cancer model (A) Schematic illustration of the AOM/DSS protocol and mouse body weight changes during the AOM/DSS treatment in Acss2^f/f^ and CEC-Acss2^−/−^ mice. (B) Gross images of the luminal side of longitudinally opened colons from control (Ctrl), Acss2^f/f^ and CEC-Acss2^−/−^ mice. Ctrl mice received no AOM/DSS treatment; (C) Quantitative data of colon weight, colon weight to body weight ratio, tumor number per mouse and total tumor volume per mouse; Ctrl *n* = 3, Acss2^f/f^*n* = 8, CEC-Acss2^−/−^*n* = 12; (D) Distribution of colon tumors with different size in Acss2^f/f^ and CEC-Acss2^−/−^ mice; (E) Acetyl-CoA concentration in colon tumor lysates; n = 5–10 each group; (F) Western blot analysis of colon tumor lysates; (G) Immunostaining of cleaved caspase 3 in colon tumor sections; scale bars, 100 μm. Data were presented as mean ± SD. Statistical analyses were performed by unpaired two-way ANOVA or Student’s *t* test.

To validate the role of ACSS2 in CRC growth, we next examined the effect of *Acss2* deletion using an azoxymethane (AOM)/dextran sodium sulfate (DSS)-induced colitis-associated colorectal cancer (CAC) model and an *Apc*-mutated sporadic colon cancer model. The AOM/DSS-induced mouse CRC model recapitulates the features of inflammation-associated colonic tumorigenesis, which is generated by treating the mice with a single dose of AOM followed by 2-3 cycles of DSS supplementation in the drinking water.^24^^,^^25^ We generated *Acss2*^f/f^;*Cdx2*-Cre (CEC-*Acss2*^−/−^) mice to delete *Acss2* from colonic epithelial cells (CECs), as the *Cdx2* promoter drives Cre expression in epithelial cells from distal ileum to distal colon.^26^ We subjected *Acss2*^f/f^ (as control) and CEC-*Acss2*^−/−^ mice to the AOM/DSS procedure to induce colonic tumorigenesis (Figure 4A). Although there were no differences in body weight changes between these two genotypes during the AOM/DSS treatment (Figure 4A), CEC-*Acss2*^−/−^ mice had a marked reduction in colon tumor load compared with the *Acss2*^f/f^ control (Figure 4B). Quantitatively, CEC-*Acss2*^−/−^ mice showed a significantly lower colon weight, reduced colon/body weight ratio, fewer tumors per mouse, and smaller total tumor volume per mouse (Figure 4C). Importantly, CEC-*Acss2*^−/−^ mice had fewer large (>3 mm) tumors in the colon per mouse (Figure 4D). As ACSS2 catalyzes the conversion of acetate to acetyl-CoA, we measured the acetyl-CoA content in the tumor lysates and found that the *Acss2*^−/−^ tumors had significantly lower acetyl-CoA concentrations than the *Acss2*^f/f^ tumors (Figure 4E). Western blot analysis showed a marked increase in activated caspase 3 in the *Acss2*^−/−^ tumors (Figure 4F), which is validated by immunostaining analysis (Figure 4G). These data indicate that ACSS2 plays a critical role in colon tumor progression and support a mechanism involving blockade of tumor cell apoptosis.

**Figure 5:**
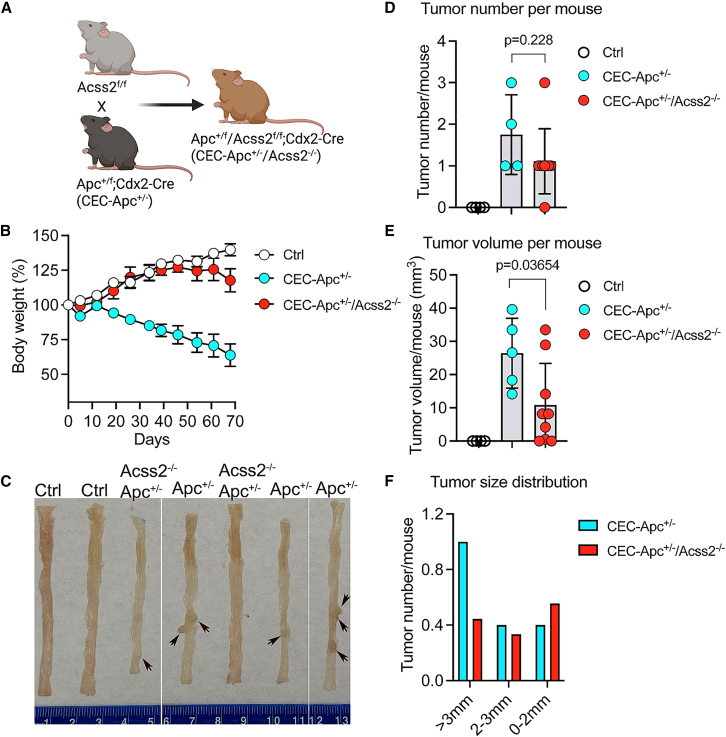
Genetic ablation of the *Acss2* gene reduces colon tumor loads in a sporadic colon cancer model (A) Schematic illustration of mouse crossing for the generation of CEC-Apc^+/-^/Acss2^−/−^ mice; (B) Mouse body weight changes after birth; (C) Gross images of the luminal side of longitudinally opened colons from control (Ctrl), CEC-Apc^+/-^ and CEC-Apc^+/-^/Acss2^−/−^ mice. Ctrl mice are wild-type mice; (D) Colon tumor number per mouse; (E) Total tumor volume per mouse; (F) Distribution of colon tumors with different sizes in CEC-Apc^+/-^ and CEC-Apc^+/-^/Acss2^−/−^ mice. Ctrl *n* = 4, CEC-Apc^+/-^ n = 4–5, CEC-Apc^+/-^/Acss2^−/−^*n* = 9; data were presented as mean ± SD. Statistical analyses were performed by unpaired two-way ANOVA.

Somatic adenomatous polyposis coli (*APC*) mutations are the most common mutations in human CRC, and *Apc* inactivation in mice results in colonic tumorigenesis^26^ that recapitulates the features of sporadic colorectal cancer.^27^ We have reported that *Apc*^+/f^;*Cdx2*-Cre mice that carry a heterozygous deletion of exon 14 of the *Apc* allele from colonic epithelial cells develop adenomas in the colon following the loss of heterozygosity.^28^ To assess the role of ACSS2 in sporadic colon tumor development, we crossed *Acss2*^f/f^ and *Apc*^+/f^;*Cdx2*-Cre (CEC-*Apc*^+/-^) mice to generate compound mutant *Apc*^+/f^/*Acss2*^f/f^;*Cdx2*-Cre (CEC-*Apc*^*+/-*^/*Acss2*^−/−^) mice (Figure 5A). These mice are all on C57BL/6 background. CEC-*Apc*^+/-^ mice gradually lost weight over time (Figure 5B), and developed very prominent tumors in the colon (Figure 5C). In contrast, CEC-*Apc*^*+/-*^/*Acss2*^−/−^ mice gained weight comparable to non-tumor bearing mice and only started to lose weight at about two months of age (Figure 5B), and exhibited a marked reduction in colon tumor load (Figure 5C). Although total tumor numbers per mouse were not significantly different between these two phenotypes (Figure 5D), total tumor volume per mouse was significantly reduced in CEC-*Apc*^*+/-*^/*Acss2*^−/−^ mice (Figure 5E). Indeed, CEC-*Apc*^*+/-*^/*Acss2*^−/−^ mice had fewer large (>3 mm) tumors per mouse than CEC-*Apc*^+/-^ mice (Figure 5F), confirming a critical role for ACSS2 in the progression of colon tumors.

We further examined the role of ACSS2 using a much more aggressive CRC model that carries *Apc* homozygous deletions. We generated *Apc*^f/f^;*Cdx2*-CreERT2 and *Apc*^f/f^/*Acss2*^f/f^;*Cdx2*-CreERT2 mice, in which homozygous deletion of *Apc* gene or both *Apc* and *Acss2* genes can be induced in colonic epithelial cells, respectively, by tamoxifen (TAM) treatment (designated as CEC-*Apc*^−/−^ and CEC-*Apc*^−/−^/*Acss2*^−/−^) (Figure S1A). Following TAM treatment CEC-*Apc*^−/−^ mice rapidly lost weight in 3 weeks (Figure S1B), developed massive tumor burden in the proximal colon (Figure S1C), and all died by 51 days (Figure S1D). In comparison, CEC-*Apc*^−/−^/*Acss2*^−/−^ mice showed less weight loss, developed fewer and smaller tumors, and survived at least 10 days longer (Figures S1B–S1D). Overall, CEC-*Apc*^−/−^/*Acss2*^−/−^ mice exhibited lower tumor burden as quantified by colon weight, colon-to-body weight ratio, and tumor-covered areas on the luminal side of the colon (Figure S1E). Western blot and immunostaining data confirmed increased apoptosis in the CEC-*Apc*^−/−^/*Acss2*^−/−^ tumors compared with the CEC-*Apc*^−/−^ tumors (Figures S1F and S1G). The levels of proliferating cell nuclear antigen (PCNA), cyclin A2, β-catenin and H3K27Ac were also reduced in the CEC-*Apc*^−/−^/*Acss2*^−/−^ tumors (Figure S1F). We also confirmed in this model that acetyl-CoA levels were significantly reduced in the CEC-*Apc*^−/−^/*Acss2*^−/−^ tumors (Figure S1H). These observations provide further evidence to support the critical role of ACSS2 in colonic tumorigenesis.

### Pharmacological inhibition of ACSS2 reduces colon cancer growth

**Figure 6:**
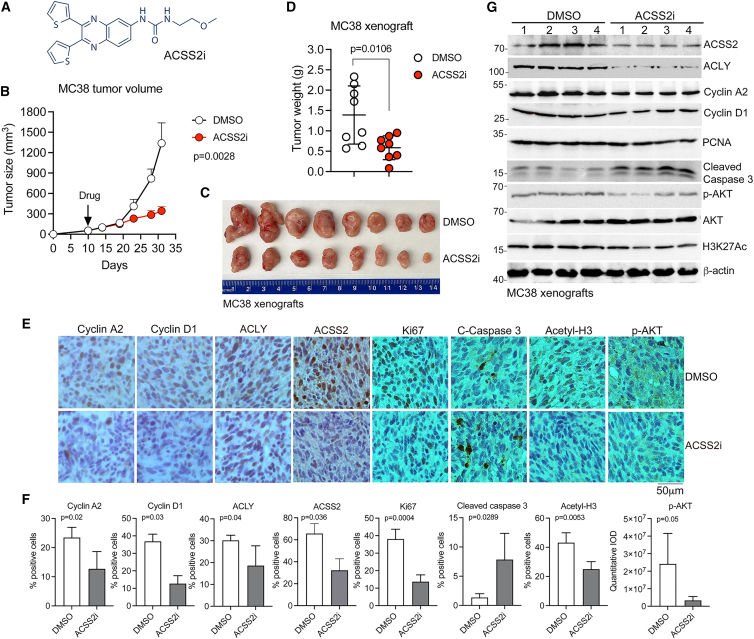
Treatment with ACSS2 inhibitor suppresses the growth of CRC allograft tumors C56BL/6 mice were subcutaneously implanted with MC38 cells. The recipient mice were treated daily with DMSO vehicle or ACSS2i (10 mg/kg daily, i.p.) on day 10 post-implantation. (A) ACSS2i chemical structure; (B) Allograft tumor growth curve; (C) Gross images of the allograft tumors; (D) Tumor weight measured at the end of the experiment; *n* = 8 each group; (E) Representative immunostaining images of the allograft tumors stained with indicated antibodies; scale bars, 50 μm. (F) Quantitative data of positively stained cells; each data point was obtained by counting positive cells from 3 to 4 independent image fields; (G) Western blot analyses of tumor lysates with indicated antibodies. Data were presented as mean ± SD. Statistical analyses were performed by two-tailed Student’s *t* test.

Our animal studies collectively suggest that ACSS2 represents an attractive therapeutic target for CRC treatment. To test this concept, we first treated MC38 cells with a small-molecule ACSS2 inhibitor (ACSS2i) (Figure 6A). ACSS2i markedly suppressed MC38 cell proliferation (Figures S2A and S2B), and cell cycle analysis revealed that ACSS2i blocks G0/G1 and G2/M phase transitions (Figures S2C and S2D). Western blot analysis showed that ACSS2i dose-dependently downregulates the expression of PCNA, cyclin A2 and Rho family-alpha serine/threonine protein kinase (AKT) phosphorylation (cell proliferation and survival markers) as well as fatty acid synthase (FASN) and 3-hydroxy-3-methylglutaryl-CoA reductase (HMGCR), two key enzymes involved in fatty acid and cholesterol biosynthesis, respectively (Figure S2E).

We then examined the effects of ACSS2i on MC38 tumor allograft growth in mice. Consistent with the *in vitro* observations, treatment with ACSS2i markedly suppressed the growth of MC38 allograft tumors (Figure 6B) and reduced the size and weight of these tumors (Figures 6C and 6D). Immunostaining and western blotting analyses of these tumors consistently showed that ACSS2i treatment down-regulated Ki67, cyclin A2, cyclin D1, and AKT phosphorylation, reduced H3K27 acetylation and induced caspase 3 cleavage (apoptosis marker) (Figures 6E–6G). Similar results were obtained from ACSS2i treatment of HCT116 xenografts, a CRC model with the human colon cancer cell line HCT116 implanted into immunocompromised Rag1^−/−^ mice (Figures S3A–S3F).

**Figure 7:**
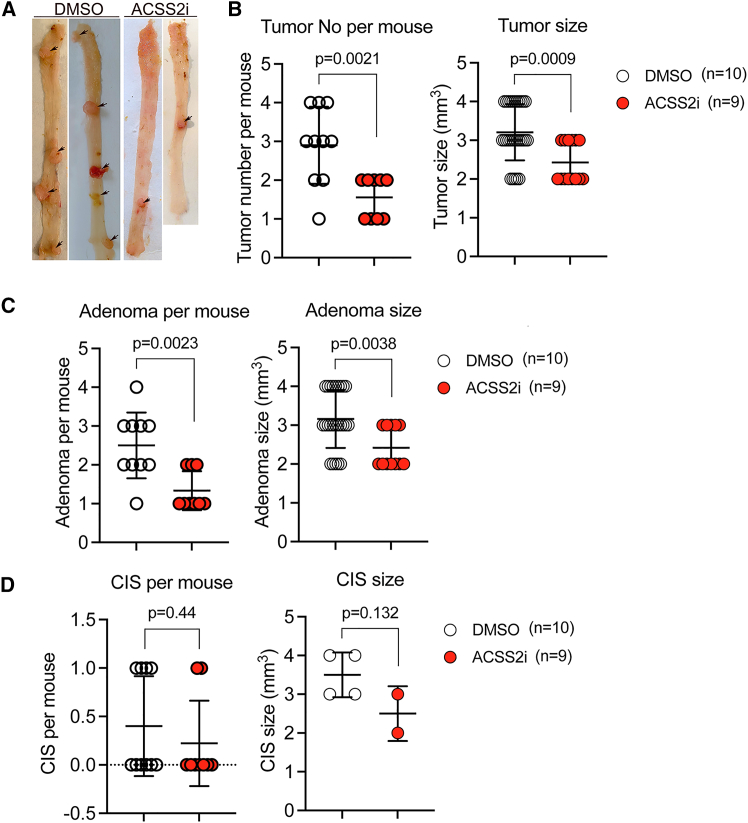
Treatment with ACSS2 inhibitor blocks the growth of *Apc*^+/-^ sporadic colon tumors (A) Gross images of the luminal side of longitudinally opened colons from CEC-Apc^+/-^ mice treated with DMSO or ACSS2i (10 mg/kg, i.p. 3 times per week). (B) Quantitative data of tumor number per mouse and tumor size in these mice treated with DMSO or ACSS2i; (C) Quantitation of adenoma number per mouse and adenoma size in these mice; (D) Quantitation of carcinoma *in situ* (CIS) per mouse and CIS size. n = 9–10 each group. Data were presented as mean ± SD. Statistical analyses were performed by two-tailed Student’s *t* test.

We further studied the anti-tumor effect of ACSS2i using the CEC-*Apc*^+/-^ sporadic colon cancer model. Treatment of CEC-*Apc*^+/-^ mice with ACSS2i resulted in a significant reduction in colon tumor burden (Figure 7A), manifested by significant decreases in tumor numbers per mouse and tumor size (Figure 7B). More detailed examination showed that ACSS2i reduces the number and size of adenomas (Figure 7C), but had no effects on the development of carcinomas *in situ* (CIS) (Figure 7D). Collectively, these observations demonstrate that ACSS2 is a druggable target for CRC therapy, and small-molecule ACSS2 inhibitors have great therapeutic potential for CRC treatment.

### Combination therapies show greater anti-tumor efficacy

**Figure 8:**
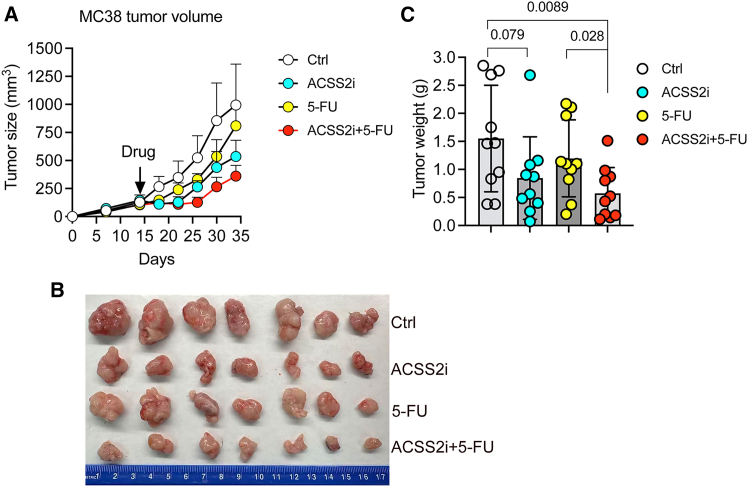
Additive efficacy of combination therapy with ACSS2i and 5-fluorouracil (5-FU) C56BL/6 mice were subcutaneously implanted with MC38 cells. The recipient mice were treated daily with ACSS2i (10 mg/kg, i.p.) alone, 5-FU (15 mg/kg, i.p.) alone, or a combination of ACSS2i and 5-FU. (A) Xenograft tumor growth curves; (B) Gross images of excised xenograft tumors; (C) Tumor weight measured at the end of the experiment; *n* = 10 each group. Data were presented as mean ± SD. Statistical analyses were performed by unpaired two-way ANOVA.

Chemotherapy is a routine treatment for advanced localized and metastatic CRC. To further explore the anti-tumor effect of ACSS2i, we tested the efficacy of a combination therapy with ACSS2i and 5-fluorouracil (5-FU) in the MC38 allograft model. 5-FU is a commonly used chemotherapy agent in human CRC. As expected, either ACSS2i or 5-FU alone was able to suppress the growth of MC38 tumors, albeit to different degrees, but the combination therapy showed additive inhibitory effects on tumor growth (Figures 8A–8C). These results suggest that combining ACSS2i with conventional chemotherapy may increase efficacy against human colon cancers.

## Discussion

In this study, we presented compelling evidence that demonstrates the requirement of ACSS2 for the growth and progression of colorectal tumors. We also established that ACSS2 can be pharmacologically targeted by small-molecule inhibitors for the treatment of colorectal cancer in animal models. Our initial observation that ACSS2 expression is up-regulated in both human and mouse CRC prompted us to investigate the role of ACSS2 in colonic tumorigenesis. By taking a loss-of-function approach, we showed that ACSS2 KD leads to a marked decrease in CRC tumor allograft and xenograft growth, and conditional genetic ablation of the *Acss2* gene from colonic epithelial cells markedly reduces colon tumor burden in both the colitis-associated colorectal cancer model and the *Apc*-mutant induced sporadic colon cancer model. Even in the very aggressive homozygous *Apc* deletion model, ACSS2 ablation is still able to significantly diminish the tumor load and prolong the survival of the mice, confirming a pivotal role of ACSS2 in CRC progression. Although the prevalence of CAC is relatively low in humans, occurring primarily in patients with inflammatory bowel disease (IBD), *APC* mutations are present in >90% of human CRC cases.^29^ As the CAC and *Apc* mutation models mimic many features of human CRC, our observations in these models have strong clinical relevance, which prompted us to explore the effect of ACSS2 blockade on CRC development. We demonstrate that a targeted inhibition of ACSS2 with a small-molecule inhibitor can substantially attenuate the progression of CRC in both allograft/xenograft and *Apc*-mutant models, confirming that ACSS2 is a druggable target that has great potential in CRC management. Moreover, the additive inhibitory effects on CRC growth seen in the ACSS2i and 5-FU combination treatment further highlight the potential value of adding ACSS2 inhibitors to conventional or emerging anti-CRC therapies in humans. Developing resistance to current chemotherapies or immunotherapies in the case of MSI-H/d-MMR CRC is not uncommon. ACSS2 inhibitors may offer the potential for novel combinations that improve outcomes with existing or emerging therapies for CRC treatment.

Our finding that ACSS2 is crucial for CRC tumor growth is consistent with the stimulatory role of ACSS2 reported in other tumors, such as breast cancer, esophageal squamous cell carcinoma, and myeloma.^3^^,^^18^^,^^30^ As a metabolic enzyme that converts acetate to acetyl-CoA, ACSS2 has been shown to promote cancer cell proliferation under metabolic stress or hypoxia through enhancing acetate utilization, H3 acetylation, and fatty acid synthesis.^3^^,^^31^ In addition, ACSS2 can stimulate cancer cell growth via direct protein interaction with other proteins, whereby providing the acetyl-CoA substrate for the acetylation of the interacting protein partners, such as IRF4, HIF-2α and phosphoribosylaminoimidazole carboxylase and phosphoribosylaminoimidazole succinocarboxamide synthase (PAICS).^8^^,^^18^^,^^32^ However, how ACSS2 affects CRC is not entirely clear in the literature. A recent study reports that ACSS2 is elevated in Kirsten rat sarcoma viral oncogene homolog (KRAS) G12V mutant CRC cells, and this mutant has greater dependence on ACSS2 for proliferative advantage compared to other mutants. ACSS2 was shown to play a critical role in early KRAS G12V adenoma development, not in KRAS G12D adenomas, suggesting mutation-specific metabolic reprogramming in KRAS-driven CRC.^33^ In contrast, another report shows that ACSS2 expression is significantly decreased in human CRC compared with corresponding normal mucosa tissues, and ACSS2 down-regulation is believed to be a metabolic hallmark of tumor progression in colorectal carcinoma.^34^ The data from our study strongly support the notion that ACSS2 promotes CRC growth and progression. Our data show that ACSS2 up-regulation in colorectal tumors renders the tumor cells more resistant to stress-induced apoptosis, while concomitantly blocking the recruitment of immune cells, including CD8^+^ T cells and macrophages that have anti-tumor capacities, into the tumor microenvironment. Together, these actions are predicted to create favorable conditions for tumor progression. However, the exact molecular mechanisms underlying these activities remain unclear. Given that ACSS2 KD leads to decreases in ACSS2 nuclear translocation and H3 acetylation, we speculate that an epigenetic mechanism is involved in ACSS2-mediated tumor progression.

Another controversy in the literature is exactly how acetate influences colorectal cancer. Colon cancer cells can uptake extracellular acetate via transporters MCT1/SMCT1,^35^ but acetate has been reported to induce growth arrest in colon cancer cells through the modulation of mitochondrial function^36^ or induce apoptosis via alteration of lysosomal membrane permeabilization.^37^ Another study showed that butyrate, not acetate, can cause cell growth arrest and differentiation of human colon cancer cells via histone Hyperacetylation.^38^ A similar controversy is seen in the case of glioblastoma, where acetate is believed to be a bioenergetic substrate for cancer growth,^17^ yet acetate supplementation can induce glioblastoma cell growth arrest.^39^ Acetate is the most abundant short-chain fatty acids (SCFAs) in the colon, accounting for about 60% of the luminal SCFAs^40^; as such, it is logical to speculate that CRC can conveniently use the high concentration of luminal acetate to support rapid tumor growth. However, our data show that ACSS2 KD or ACSS2i treatment induces excessive apoptosis and suppresses colon cancer cell proliferation in the absence of extracellular acetate, suggesting that the acetate that supports colon cancer cell growth is likely from an intracellular, rather than an extracellular source. Intracellular acetate can be released from histone deacetylation, and nuclear ACSS2 is known to reuse this acetate to maintain histone acetylation during hypoxia and nutritional stress.^7^ Intracellular acetate can also be generated from acetyl-CoA by acyl-CoA thioesterase 12,^41^ or from a combination of acetyl-CoA and aspartate to N-acetylaspartate in the mitochondria, which is converted to aspartate and acetate by aspartoacylase in the cytosol.^42^^,^^43^ Thus, the source of acetate used as an ACSS2 substrate to support CRC growth requires further investigation.

There were almost two million new cases of CRC and one million deaths worldwide in 2020, causing enormous socio-economic and health care burdens.^44^ As such, more effective therapeutic strategies are urgently needed for CRC management. Given the distinct metabolism of cancer cells, targeting cancer metabolism has emerged as a new therapeutic strategy.^45^ Our study suggests that targeting ACSS2 with small-molecule inhibitors is a promising strategy for CRC treatment, which lays the foundation for clinical trials in patients with CRC. In fact, an ACSS2 inhibitor (MTB-9655) has been used in a phase I clinical trial in patients with advanced solid tumors, and the drug is well tolerated with a predictable safety profile.^46^ Future research should focus on the identification of new ACSS2 inhibitors with few side effects and better efficacy. Given the synergistic outcome from the ACSS2i and 5-FU combination therapy, future studies may also test the combination of ACSS2 inhibitors and immune checkpoint inhibitors.^47^ As ACSS2 inhibition can increase immune cell recruitment in the tumors, which may convert the cold tumor microenvironment (immune checkpoint inhibitor unresponsive) in most CRC to immune susceptible ones as occurs in microsatellite unstable CRC, its combination with immunotherapy might achieve a better anti-tumor efficacy.

### Limitations of the study

There are several limitations in this study. Although our data suggest that ACSS2 promotes colorectal tumor progression by inhibiting tumor apoptosis under nutritional and hypoxic stresses and preventing the recruitment of immune cells into the tumor microenvironment, the exact molecular mechanism remains to be determined. Based on our data, we speculate that ACSS2 depletion leads to increased DNA damage that triggers activation of the p53-dependent apoptotic pathway, and cell apoptosis subsequently triggers chemotaxis and immune activation, leading to disruption of tumor cells. The molecular basis underlying these speculations needs further experimental validation. Moreover, we did not exclude the potential off-target effects of the ACSS2 inhibitor used in the study. In theory, effects unrelated to ACSS2 may also slow down tumor growth. Further studies are needed to exclude this possibility.

## Resource availability

### Lead contact

Further information and requests concerning resources and reagents should be directed to and will be fulfilled by the lead contact, Yan Chun Li (cyan@bsd.uchicago.edu).

### Materials availability

Lentiviral constructs and mouse lines generated in this study will be available from the corresponding author upon request.

### Data and code availability

• The RNA-seq data generated in this study are available from GEO under the accession number GSE315027.
• This paper does not report original code.
• Any additional information required to reanalyze the data reported in this paper is available from the lead contact upon request.

## Acknowledgments

This work was supported in part by Cancer Research Foundation Fletcher Scholar Award AWD103497, 10.13039/100018785GI Research Foundation, and 10.13039/100000002NIH (grant no. R01DK138355) (to Y.C.L.).

## Author contributions

Conceptualization Y.C.L.; methodology and investigation L.W., U.D., and C.M.; formal analysis L.W., L.G., C.M., Y.C.L.; writing—original draft L.W. and Y.C.L.; writing—review and editing A.S., H.L., M.B., and Y.C.L.; fund acquisition Y.C.L.; resources R.S., W.H., J.D., A.S., H.L., and M.B.; supervision Y.C.L.

## Declaration of interests

The authors declare no competing interests.

## STAR★Methods

### Key resources table

**Table:** 

REAGENT or RESOURCE	SOURCE	IDENTIFIER
**Antibodies**		

Anti-p53	ProteinTech	Cat#: 10442-1-AP, RRID:AB_2206609
Anti-CD8	Cell Signaling Technology	Cat#:98941, *RRID*:AB_2756376
Anti-F4/80	ProteinTech	Cat#:28463-1-ap, RRID: AB_2881149
Anti-ACSS1	ProteinTech	Cat#: 17138-1-AP, RRID:AB_2289182
Anti-ACSS2	Cell Signaling Technology	Cat#: 3658S, RRID:AB_2222710
Anti-PCNA	ProteinTech	Cat#: 60097-1-Ig, RRID:AB_2236728
Anti-cleaved-caspase-3	Cell Signaling Technology	Cat#: 9661, RRID:AB_2341188
Anti-Cyclin D1	ProteinTech	Cat#: 60186-1-1g, *RRID*: AB_10793718
Anti-FASN	Cell Signaling Technology	Cat#: 3180, RRID:AB_2100796
Anti-HIF-2α	Cell Signaling Technology	Cat#: 48085, RRID:AB_3698324
Anti-Lamin B1	ProteinTech	Cat#: 12987-1-AP, RRID:AB_2136290
Anti-Tubulin	ProteinTech	Cat#: 66031-1-Ig, RRID: AB_11042766
Anti-Cyclin A2	ProteinTech	Cat#: 18202-1-AP, RRID: AB_10597084
Anti-H3K27ac	Milipore	Cat#: MABE647, RRID: AB_2893037
Anti-p-Histone H2AX	Cell Signaling Technology	Cat#: 9718S, RRID:AB_2118009
Anti-*p*-ACSS2-Ser659	Signalway antibody	Cat#: 58003
Anti-beta-catenin	ProteinTech	Cat#: 51067-2-ap, RRID:AB_2086128
Anti-Acetyl-Histone H3	Cell Signaling Technology	Cat#: 9677S, RRID:AB_1147653
Anti-ACLY	Abcam	Cat#: ab40793, RRID:AB_722533
Anti-Ki67	invitrogen	Cat#: Ma5-14520, RRID:AB_10979488
Anti-HMGCR	ProteinTech	Cat#: 30469-1-ap, RRID:AB_3086329
Anti-β-Actin	Santa Cruz Biotechnology	Cat#: sc-47778, RRID:AB_626632
Anti-mouse IgG-HRP	Santa Cruz Biotechnology	Cat#: sc-516102, RRID:AB_2687626
Anti-rabbit IgG-HRP	Santa Cruz Biotechnology	Cat#: sc-2357, RRID:AB_628497
Anti-mouse IgG-HRP	Cell Signaling Technology	Cat#: 7076, RRID:AB_330924
Anti-rabbit IgG-HRP	Cell Signaling Technology	Cat#: 7074, RRID: AB_2099233
Anti-Histone H3	Millipore	Cat#: 06-755, RRID: AB_2118461

**Chemicals, Peptides and Recombinant Proteins**		

(Z)-4-Hydroxytamoxifen	Sigma-Aldrich	Cat#: H7904
Tamoxifen	Sigma-Aldrich	Cat#: T5648
Neomycin	Sigma-Aldrich	Cat#: N1876
Ampicillin	Sigma-Aldrich	Cat#: A9518
2-deoxy-D-glucose (2-DG)	Sigma-Aldrich	Cat#: D6134
5-fluorouracil (5-FU)	Millipore	Cat#: F6627
ACSS2 inhibitor	MedChemExpress	Cat#: 508186-14-9
Azoxymethane (AOM)	Sigma-Aldrich	Cat#: A5486
Dextran sodium sulfate (DSS)	Thermo Scientific	Cat#: J63606.22
Matrigel Matrix	Corning	Cat#: A3011
Propidium iodide	Alfa Aesar	Cat#: J66584

**Critical Commercial Assays**		

KAPA mRNA hyperPrep kit	Roche	Cat#: Kr1352
KAPA unique dual-indexed adapter kit	Roche	Cat#: Kr1736
Dynabeads mRNA Purification Kit	ThermoFisher	Cat#: 61006
Annexin V Apoptosis Detection Kit	BD Biosciences	Cat#: 559763
Acetyl-CoA assay kit	Abcam	Cat#: AB87564

**Deposited data**		

RNA-seq	This work	GEO: GSE315027

**Experimental Models: Cell Lines**		

MC38	Kerafast Co	Cat#: CVCL_B288
HCT116	ATCC	Cat#: CCL-247

**Experimental Models: Organisms/Strains**		

*Acss2*^flox/flox^ mice (C57BL/6-Acss2tm1.2 mrl)	Taconic Biosciences	Stock No: 10365
CDX2-Cre transgenic mice (B6.Cg-Tg(CDX2-cre)101Erf/J)	Jackson Laboratory	Stock No: 009350
CDX2-CreERT2 transgenic mice (B6.Cg-Tg(CDX2-Cre/ERT2)752Erf/J)	Jackson Laboratory	Stock No: 022390
*Apc*^flox/flox^ mice (C57BL/6 background)	NCI Mouse Repository	Stock No: 01XAA

**Recombinant DNA**		

pLV[shRNA]-EGFP:T2A:Neo-U6>mAcss2[shRNA#1]	Vector Builder	VB240322-1456ujh
pLV[shRNA]-EGFP:T2A:Neo-U6>mAcss2[shRNA#2]	Vector Builder	VB240322-1458kcr
pLV[shRNA]-EGFP:T2A:Neo-U6>Scramble	Vector Builder	VB010000-9465xev

**Software and algorithms**		

ImageJ	NIH	https://imagej.nih.gov/ij/
Image-pro plus	Media Cybernetics, Inc	https://mediacy.com/
Prism 9.1.0	GraphPad	https://graphpad-prism.software.informer.com/#google_vignette
R software v4.3.3	R Core team	https://www.r-project.org/
DESeq2	Bioconductor	https://bioconductor.org/packages/DESeq2/

### Experimental model and study participant details

#### Animals

*Apc*^f/f^ mice were obtained from NCI Mouse Repository (Stock #01XAA). *Acss2*^f/f^ mice (C57BL/6-Acss2tm1.2 mrl) were obtained from Taconic Biosciences (Stock #10365). *Cdx2*-Cre transgenic mice (B6.Cg-Tg(CDX2-cre)101Erf/J; Stock # 009350) and Cdx2-CreERT2 transgenic mice (B6.Cg-Tg(CDX2-Cre/ERT2)752Erf/J; Stock # 022390) were purchased from Jackson Laboratory. *Apc*^+/f^;*Cdx2*-Cre, *Acss2*^f/f^;*Cdx2*-Cre, *Apc*^+/f^/*Acss2*^f/f^;*Cdx2*-Cre, *Apc*^f/f^;*Cdx2*-CreERT2, and *Apc*^f/f^/*Acss2*^f/f^;*Cdx2*-CreERT2 mice were obtained through interbreeding. All mice were on C57BL/6 background. Both male and female were used in experiments, and littermates were used in the study. Animals were randomly assigned to experimental groups. To activate CreERT2 recombinase activity, mice were injected intraperitoneally with three doses of tamoxifen (TAM) (one dose = 0.1mL at 10 mg/mL) on three consecutive days. Injection of corn oil (Oil) served as control. All mice were housed at 25°C and maintained in a 12h/12h light/dark cycle. To ensure that the animals are subject to minimal discomfort and humane treatment, all procedures were performed under anesthesia when the animals were no longer responsive to painful stimuli. Ketamine (90-120 mg/kg body weight) and xylazine (5-10 mg/kg body weight) were used for rapid procedures such as subcutaneous tumor cell injection. Euthanasia were performed by CO_2_ inhalation, and death was confirmed by cervical dislocation. For tissue collection, animals were anesthetized with xylazine and ketamine and were no longer responsive to painful stimuli prior to sacrifice by exsanguination. All animal study procedures were approved by the Institutional Animal Care and Use Committee (IACUC) at the University of Chicago, protocol # 71525, and followed institutional guidelines.

#### Cell culture

MC38 cell line (CVCL_B288) was purchased from Kerafast Co. HCT116 cell line (CRL-(247) was purchased from ATCC. MC38 and HCT116 cells were grown in DMEM supplemented with 10% heat-inactivated fetal bovine serum (FBS), 2 mM glutamine, 1 mM sodium pyruvate, 100 U/mL penicillin and 100 μg/mL streptomycin at 37°C and 5% CO_2_. Cell identities were validated by ATCC and tested negative for mycoplasma by IDEXX BioAnalytics.

### Method details

#### Crystal violet staining

Cells were grown in 12- or 6-well plates, and treated with DMSO or 10 μM ACSS2i (MedChemExpress) for 1, 2 or 3 days. Following treatment, the culture medium was removed, and the cells were fixed in 10% neutral formalin solution for 15 min. The fixed cells were stained in 0.02% aqueous solution of crystal violet for 30 min. After washing with PBS, the stained cells were solubilized with 70% ethanol and absorbance quantified at 590 nm using a microplate reader.

#### Cell cycle analysis

Cells were treated with 0, 5 and 10 μM ACSS2i for 24 h, and stained with 20 μg/mL propidium iodide. The cells were analyzed by FACS to assess cell cycle phases.

#### Lentiviral constructs

Lentiviral constructs carrying an shRNA were generated on pLV[shRNA]-EGFP:T2A:Neo-U6 lentiviral backbone (VectorBuilder). Mouse *Acss2*-specific shRNA target sequences are 5′- GTGTGTCAGTTCAGCAATGTT-3’ (shRNA1) and 5′-CGGTTTGAGACCACCTACTTT-3’ (shRNA2). All lentiviruses were produced by transfecting HEK293T packaging cells with a titer of ∼10^7^^,^^8^ pfu/mL.

#### ACSS2 knockdown

MC38 cells were transduced with scramble RNA control (Ctrl) lentivirus or lentivirus carrying shRNA1 or shRNA2 at 10 MOI in the presence of polybrene (6 μg/mL). Following incubation at 37°C overnight, the virus was washed out and the cells cultured in the presence of G418 (0.5 mg/mL). Stable clones were obtained after one to two-week selection and ACSS2 protein levels in the cells were quantified by Western blotting.

#### Measurement of apoptosis

MC38 stably transduced with Ctrl or shRNA1 lentivirus were exposed to a medium containing 1% FBS and 10 mM 2-deoxy-D-glucose (2-DG) for 2 days. Cell apoptosis was assessed by annexin V staining using an Annexin V Apoptosis Detection Kits (BD Biosciences) and quantified by FACS.

#### Allograft and xenograft models

MC38 tumor allografts were produced in C57BL/6 mice and HCT116 xenografts generated in *Rag1*^−/−^ mice. Briefly, mice were lightly restrained and anesthetized with isoflurane and the flanks shaved with an electric shear. Cells suspended in PBS (1 × 10^6^/0.2 mL/site) were injected subcutaneously in both flanks of each mouse. To study the effects of ACSS2 inhibition, some mice were treated with DMSO (as control) or ACSS2i at 10 mg/kg daily via intraperitoneal injection one week after tumor cell transplantation. For combination therapy, some mice were treated with DMSO, ACSS2i (10 mg/kg) only, 5-FU (15 mg/kg) only or both ACSS2i (10 mg/kg) and 5-FU (15 mg/kg) daily via intraperitoneal injection. All animals were euthanized and tumors excised when the subcutaneous tumors reached 1.5 cm^3^ in combined tumor size. Tumors were resected, photographed, weighed and subjected to histological and biochemical analyses. The investigators were not blinded during the measurements.

#### AOM/DSS colon cancer model

The procedure for AOM/DSS-induced colonic tumorigenesis was performed as described previously.^48^ Briefly, *Acss2*^f/f^ and *Acss2*^f/f^;*Cdx2*-Cre (CEC-*Acss2*^−/−^) mice were treated with one dose of AOM (10 mg/kg) by i.p injection. After one week, these mice were placed on drinking water containing 2.5% DSS for 7 days, followed by regular drinking water for 14 days. This DSS water/regular water cycle was repeated for two more times. Mouse body weight was measured biweekly. After mouse sacrifice the colons were harvested immediately, opened longitudinally, cleaned by ice-cold PBS wash and photographed. Tumor numbers were counted and tumor size measured. The tumors were excised and subjected to histological and biochemical analyses. The investigators were not blinded during the measurements.

#### APC mutation colon cancer models

*Apc*^+/f^;*Cdx2*-Cre (CEC-*Apc*^+/-^) mice develop spontaneous adenomas in the colon.^28^ To assess the effect of ACSS2i on sporadic colon tumor development, CEC-*Apc*^+/-^ mice were treated with ACSS2i (10 mg/kg, i.p.) 3 times per week. To assess the effect of ACSS2 depletion on colon tumor development, *Apc*^+/f^;*Cdx2*-Cre and *Apc*^+/f^/*Acss2*^f/f^;Cdx2-Cre mice were compared, and *Apc*^f/f^;*Cdx2*-CreERT2 and *Apc*^f/f^/*Acss2*^f/f^;*Cdx2*-CreERT2 mice were compared, following TAM treatment (1 mg/mouse, daily for 3 consecutive days). Body weight was measured weekly, and tumor size were monitored by colonoscopy. After the mice were euthanized, the colons collected immediately. The colons were opened longitudinally, and the luminal surface photographed and inspected to record the size, number and distribution of tumors. Visible tumors were excised and the remaining colon processed using the “Swiss roll” technique^49^ for global histological assessment by hematoxylin and eosin (H&E) staining. For the CEC-*Apc*^−/−^ model, the colon weight and the luminal surface area covered by tumors were measured. Excised tumor samples were subjected to histological and biochemical analyses. The investigators were not blinded during the measurements.

#### Histology and immunohistochemical staining

Tumor allografts/xenografts and excised colon tumors were fixed overnight in 4% formaldehyde made in PBS (pH 7.(2) at room temperature. Colons were opened longitudinally from cecum to rectum and “Swiss rolls”^50^ prepared for fixation overnight. Fixed samples were processed, embedded in paraffin wax and cut into 4 μm sections. The sections were stained with H&E for routine structural examination. For immunohistochemical staining, sections were boiled in 10 mM sodium citrate (pH 6.0) for 10-15 min for antigen retrieval before being stained with primary antibodies. After washes, the sections were incubated with horseradish peroxidase (HRP)-conjugated secondary antibodies and the antigens were visualized by incubating with 3,3′-diaminobenzidine (DAB) as substrate. Slides were examined under a light microscope and images photographed. Immunostaining was quantified using Image Pro Plus. The investigators were not blinded during the measurements.

#### Subcellular fractionation

Nuclear, cytosolic and mitochondrial fractions were prepared based on published methods.^51^ Briefly, colonic epithelial cells or organoids were washed with cold PBS and resuspended in STM buffer (250 mM sucrose, 50 mM Tris–HCl, 5 mM MgCl_2_, protease and phosphatase inhibitor cocktails), followed by 1 min homogenization. The broken cells were then centrifuged for 15 min at 800 × g and 4°C to precipitate the nuclear fraction. The supernatant containing mitochondrial and cytosolic fractions was further centrifuged at 11,000 × g for 10 min. After centrifugation, the supernatant was precipitated in acetone at -20°C for 1 h and centrifuged at 12,000 × g for 5 min to collect the cytosolic fraction, and the pellet was resuspended in STM buffer and centrifuged at 11,000 × g for 10 min to collect the mitochondrial fraction.

#### Western blotting

Tissue and cell samples were homogenized in Laemmli buffer. Protein concentration was determined using a Bio-Rad DC RC protein assay kit. Protein lysates were separated by SDS-PAGE and then electroblotted onto Immobilon-P membranes (MilliporeSigma). The membranes were blotted with primary antibodies purchased commercially, followed by incubation with horseradish peroxidase-conjugated secondary antibody. Protein bands were visualized by chemiluminescence using an ECL Western Blot Substrate Kit (ThermoFisher). Detailed Western blot procedures were described previously.^52^ Primary antibodies used for Western blot analyses are listed in the Key Resources Table.

#### Bulk RNA-seq

Total RNA was extracted using TRIzol Reagent (ThermoFisher). RNA-seq libraries were prepared using a KAPA RNA HyperPrep Kit (Roche) according to the manufacturer’s instruction. The libraries were sequenced using an Illumina NovaSeqX System with single end 50-bp reads. Sequencing raw data were preprocessed using trim_galore v0.6.6, and reads were mapped by STAR^53^ v2.7.9a against Gencode GRCm39 (release M27) reference genome. The gene level read counts were obtained using FeatureCount via Rsubread 2.6.1. Differential expression was analyzed using R v4.3.3 and edgeR package. *p*-values were adjusted for multiple testing using the false discovery rate (FDR) correction of Benjamini and Hochberg.^54^ Genes with significant differential expression were determined based on an FDR threshold of 5% (0.05). Analyses of Reactome pathway enrichment and GO (Gene Ontology) biological process enrichment of the differentially expressed genes (DEGs) were accomplished using R package clusterProfiler.^55^

### Quantification and statistical analysis

Data were presented as means ± SD. Most experiments were repeated at least twice. All bioinformatic analyses were conducted using samples of biological triplicates. Statistical analyses were performed using GraphPad Prism Version 9.1.0. For two group comparisons unpaired two-tailed Student’s *t* test was used, and for three or more group comparisons ordinary one-way or two-way analysis of variance (ANOVA) was performed. Animal survival rates were estimated by the Kaplan-Meier method and groups were analyzed by the log rank test. *p* values <0.05 were considered statistically significant.
