# Supplementary material for: ACSS2 is required for colorectal cancer progression and a druggable target for colorectal cancer treatment
Source: iScience. 2026 Jul 21;29(8):116860. doi: 10.1016/j.isci.2026.116860 (PMC13392868; doi:10.1016/j.isci.2026.116860)
Supplement: Document S1. Figures S1–S3 [file mmc1.pdf]

iScience, Volume *29*

Supplemental information

ACSS2 is required for colorectal cancer progression and a druggable target for colorectal cancer treatment

Lei Wang, Urszula Dougherty, Wenliang He, Lu Gao, Caleb Muefong, Rajesh Sarkar, Jie Du, Ardaman Shergill, Hening Lin, Marc Bissonnette, and Yan Chun Li

SUPPLEMENTAL FIGURES AND LEGENDS

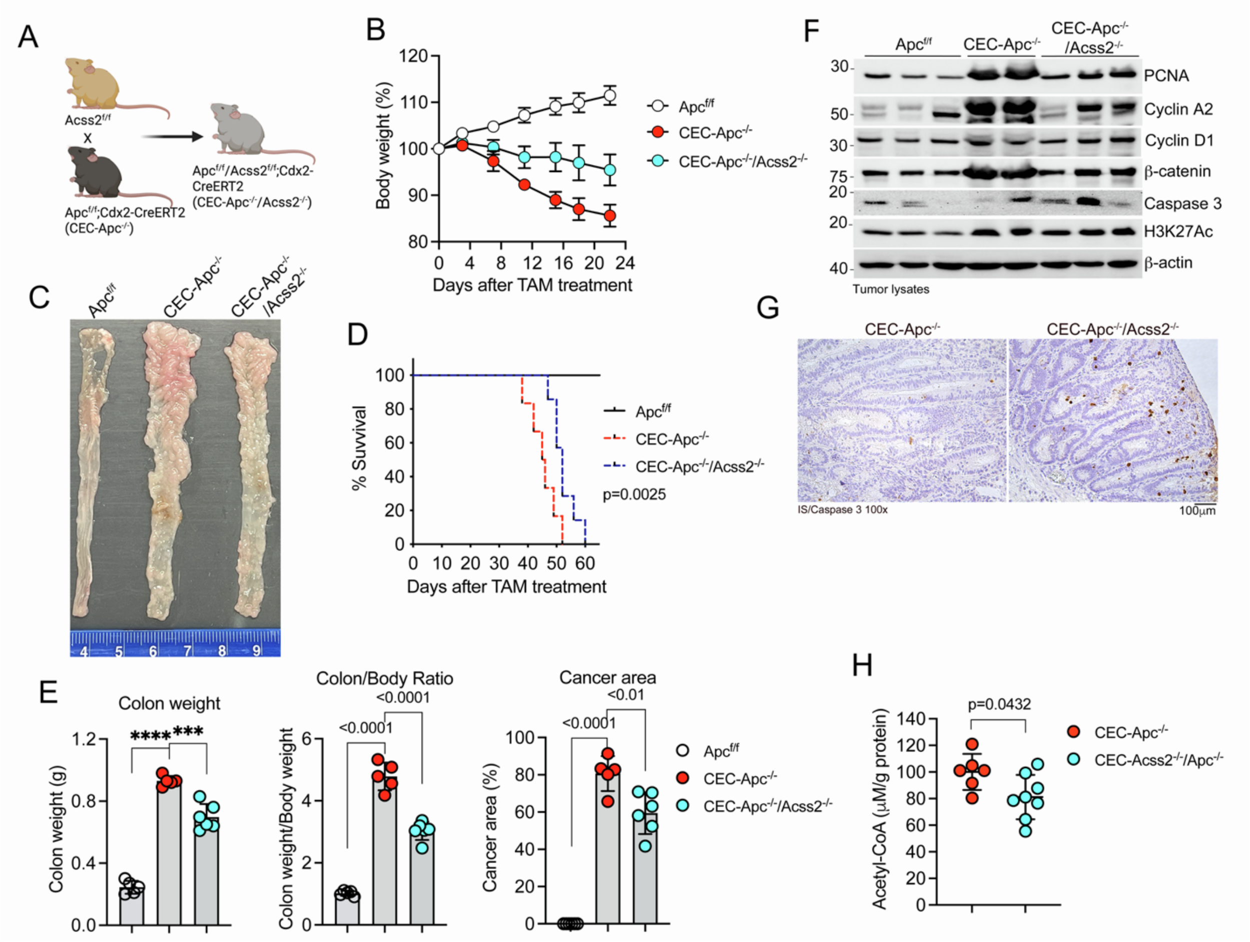

Figure S1. Genetic deletion of *Acss2* gene reduces colon tumor burden in the aggressive homozygous *Apc* mutant CRC model, Related to Figure 5.

(A) Schematic illustration of mouse crossing; (B) Mouse body weight changes following tamoxifen (TAM) treatment; (C) Luminal surface images of longitudinally opened colons; (D) Mouse survival curves; (E) Quantitative data for colon weight, colon to body weight ratio and cancer area on the luminal surface; (F) Western blot analysis of colon tumor lysates; (G) Immunostaining for cleaved caspase 3 on colon tumor sections; Scale bar: 100 μm. (H) Acetyl-CoA concentration in colon tumor lysates. Apc$^{f/f}$ n=4, CEC-Apc$^{-/-}$ n=5, CEC-Apc$^{-/-}$/Acss2$^{-/-}$ n=5; Data were presented as mean ± SD. Statistical analyses were performed by unpaired two-way ANOVA or two-tailed Student's t test. The Kaplan-Meier curves in (D) were analyzed by the log-rank test.

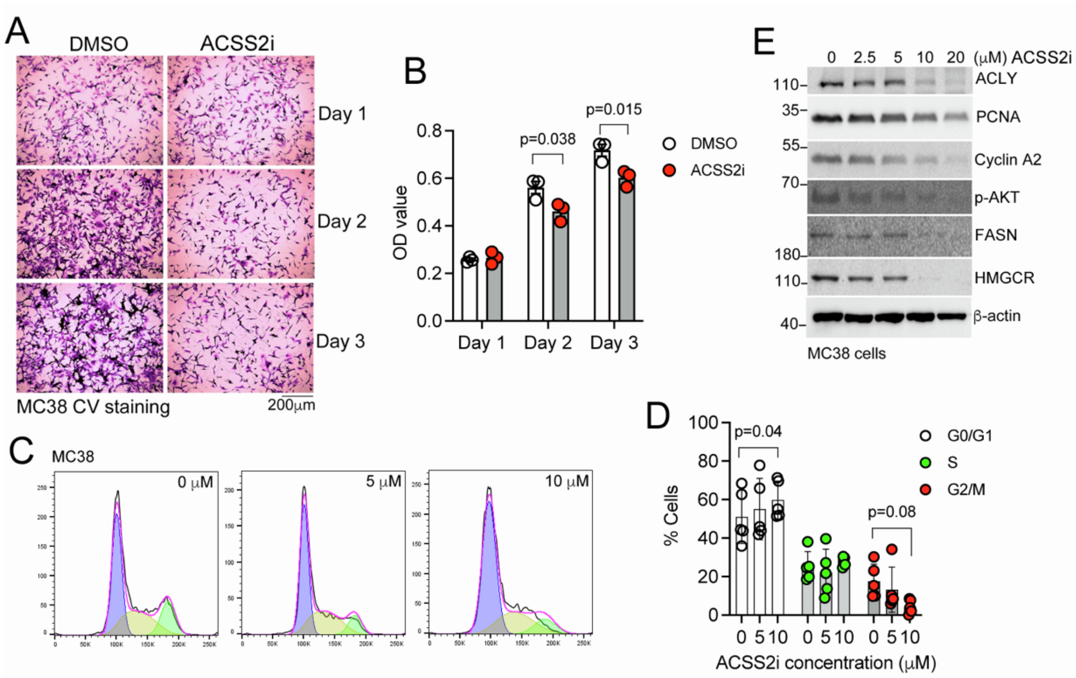

Figure S2. ACSS2 inhibitor suppresses colon cancer cell proliferation *in vitro*, Related to Figures 6 and 7.

(A,B) MC38 cell cultures were treated with DMSO vehicle or ACSS2i (10 μM) for 1, 2 or 3 days. The cells were stained with crystal violet (A), and the amount of crystal violet was quantified by measuring the absorbance at 590 nm (B); n=3 each group; (C,D) MC38 cells were treated with 0, 5 or 10 μM ACSS2i for 24 hours. The Cells were stained with propidium iodide (PI), and cell cycle was analyzed by FACS. (C) FACS histograms; (D) Quantitation of cell phases; n=5 each group; (E) Western blot analysis of MC38 cell lysates treated with different doses of ACSS2i for 24 hours. Data were presented as means ± SD. Statistical analyses were performed by Student's t test or unpaired two-way ANOVA.

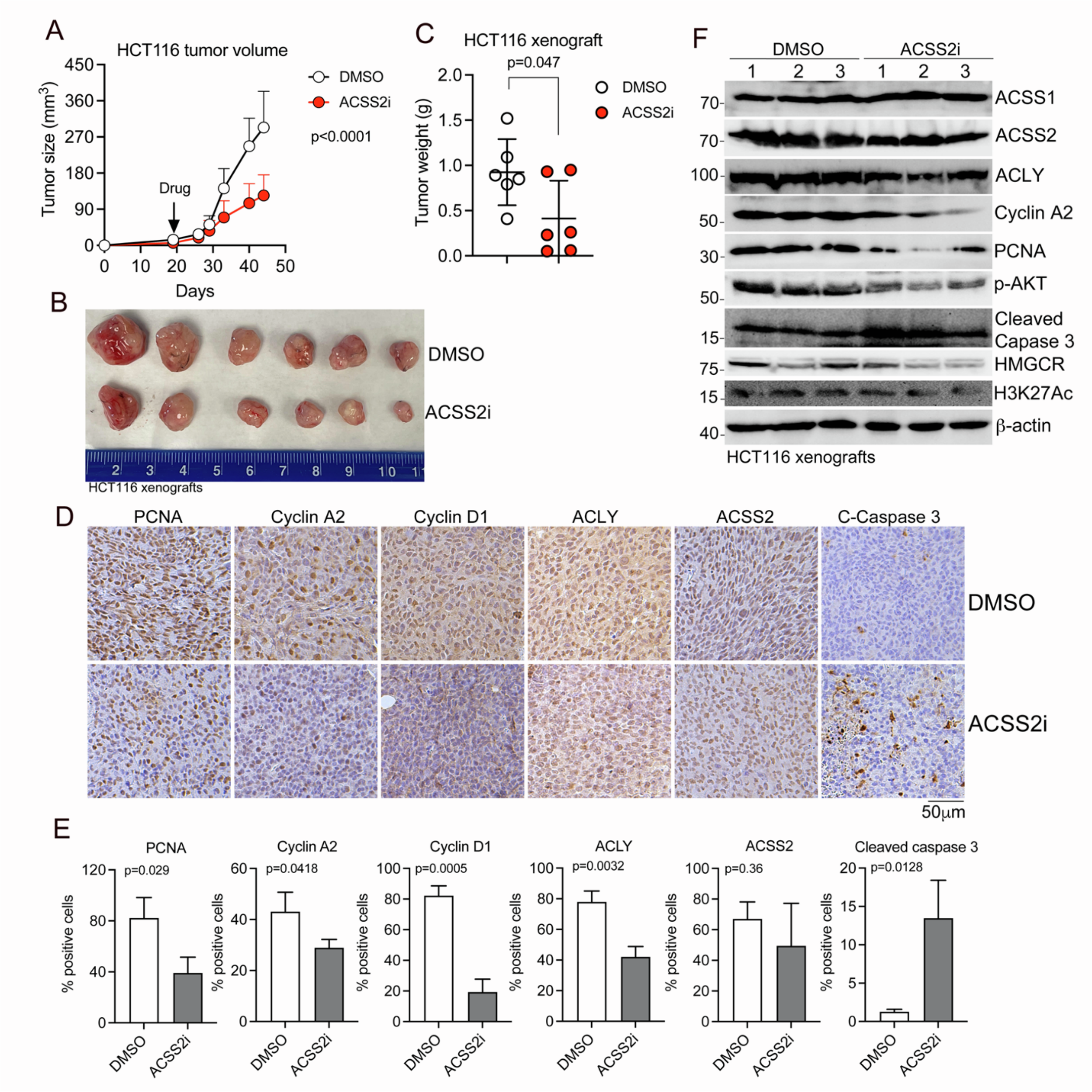

Figure S3. Treatment with ACSS2 inhibitor suppresses human CRC xenograft tumor growth, Related to Figures 6 and 7.

Rag1$^{-/-}$ mice were subcutaneously implanted with HCT116 cells. The recipient mice were treated with DMSO vehicle or ACSS2i (10 mg/kg daily, i.p.) on day 19 post implantation. (A) Xenograft tumor growth curve; (B) Gross images of isolated xenograft tumors; (C) Tumor weight measured at the end of the experiment; n=6 each group; (D,E) Representative immunostaining images of the xenograft tumors stained with antibodies indicated on the top (D) and quantitative data (E) of positively stained cells; Each data point was obtained by counting positive cells from 3-4 independent image fields; Scale bar: 50 μm. (F) Western blot analyses of tumor lysates with indicated antibodies. Data were presented as means ± SD. Statistical analyses were performed by two-tailed Student's t test.
